# Systematic Review on the Involvement of the Kynurenine Pathway in Stroke: Pre-clinical and Clinical Evidence

**DOI:** 10.3389/fneur.2019.00778

**Published:** 2019-07-19

**Authors:** Gabriela D. Colpo, Venugopal R. Venna, Louise D. McCullough, Antonio L. Teixeira

**Affiliations:** ^1^Neuropsychiatry Program, Department of Psychiatry and Behavioral Sciences, University of Texas Health Science Center at Houston, Houston, TX, United States; ^2^BRAINS Lab, Department of Neurology, University of Texas Health Science Center at Houston, Houston, TX, United States

**Keywords:** stroke, kynurenine pathway, kynurenic acid, quinolinic acid, KMO, IDO

## Abstract

**Background:** Stroke is the second leading cause of death after ischemic heart disease and the third leading cause of disability-adjusted life-years lost worldwide. There is a great need for developing more effective strategies to treat stroke and its resulting impairments. Among several neuroprotective strategies tested so far, the kynurenine pathway (KP) seems to be promising, but the evidence is still sparse.

**Methods:** Here, we performed a systematic review of preclinical and clinical studies evaluating the involvement of KP in stroke. We searched for the keywords: (“kynurenine” or “kynurenic acid” or “quinolinic acid”) AND (“ischemia” or “stroke” or “occlusion) in the electronic databases PubMed, Scopus, and Embase. A total of 1,130 papers was initially retrieved.

**Results:** After careful screening, forty-five studies were included in this systematic review, being 39 pre-clinical and six clinical studies. Despite different experimental models of cerebral ischemia, the results are concordant in implicating the KP in the pathophysiology of stroke. Preclinical evidence also suggests that treatment with kynurenine and KMO inhibitors decrease infarct size and improve behavioral and cognitive outcomes. Few studies have investigated the KP in human stroke, and results are consistent with the experimental findings that the KP is activated after stroke.

**Conclusion:** Well-designed preclinical studies addressing the expression of KP enzymes and metabolites in specific cell types and their potential effects at cellular levels alongside more clinical studies are warranted to confirm the translational potential of this pathway as a pharmacological target for stroke and related complications.

## Introduction

Stroke is clinically defined by the sudden onset of focal neurological symptoms (motor, sensory, cognitive) due to ischemia or hemorrhage in the brain. It is the second leading cause of death after ischemic heart disease and the third leading cause of disability-adjusted life-years lost worldwide (1). In the last two decades, there has been significant advance in the acute management of stroke, including the establishment of dedicated stroke inpatient units and the use of thrombolysis for eligible patients with ischemic stroke. Despite this progress, stroke-related deaths and morbidity remain a major health problem with personal and societal implications.

To address the great need of advancing stroke management, several mechanisms implicated in the pathophysiology of stroke, such as mitochondria dysfunction, glutamate-induced excitotoxicity, neuroinflammation, oxidative stress, among others, have been investigated as therapeutic targets. Among putative candidates, the kynurenine pathway (KP) received attention in the 1990's with a renewed interest recently on the wave of inflammatory-centric perspective of central nervous system diseases, including stroke (2).

The KP is the major route of tryptophan (TRP) catabolism in mammals. TRP is an essential amino acid used in the biosynthesis of proteins, being also a precursor of several bioactive molecules, such as serotonin and melatonin. Around 90% of TRP is metabolized by tryptophan 2,3-dioxygenase (TDO) into kynurenine (KYN) in the liver, with a much lower contribution of extra-hepatic KP on TRP degradation (5–10%) (3). TDO is liver specific, but two TDO variants been identified in mouse brain structures during development (4).

**Figure 1:**
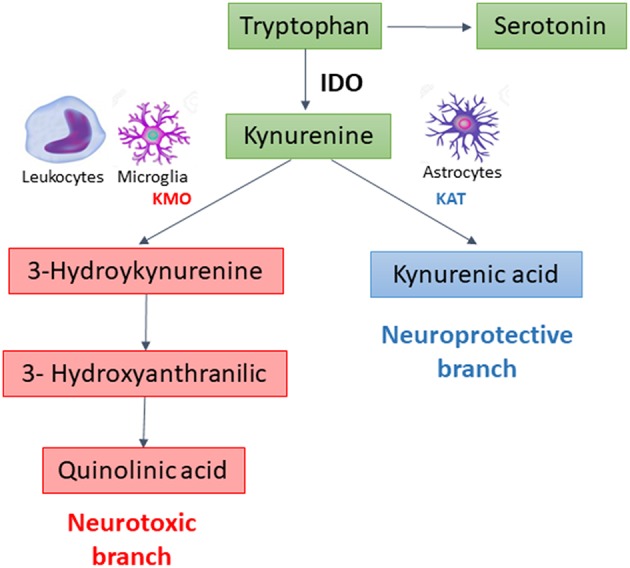
The Kynurenine Pathway (KP) of tryptophan metabolism.

In extrahepatic tissues, especially cells of the immune and central nervous systems, the KP is initiated by the degradation of TRP by indoleamine 2, 3-dioxygenase 1 (IDO), the rate limiting enzyme of the pathway. This enzyme is potently upregulated by pro-inflammatory stimuli (2). After this step, the KP branches into two major pathways–one implicated in neuroprotection, the other in neurotoxicity–that are segregated across cell types (Figure 1). Under physiological conditions, the neuroprotective branch is more active as most of kynurenine in the brain is metabolized into kynurenic acid, a NMDA and α7-nicotinic acetylcholine receptor antagonist, through the action of kynurenine aminotransferases (KATs) expressed mainly in astrocytes (5). Under inflammatory conditions, the metabolism is shifted through kynurenine-3-monooxygenase (KMO) to produce 3-hydroxykynurenine and other toxic metabolites, including quinolinic acid, a NMDA receptor agonist and an oxidative stressor (3, 6). KMO is primarily expressed in microglia, the resident immune cells in the brain, and is also expressed at high levels in peripheral immune cells such as monocytes/macrophages (7).

Besides kynurenic acid (KYNA) and quinolinic acid (QUIN), the KP produces several other biologically active metabolites, including the redox cofactors oxidized nicotinamide adenine dinucleotide (NAD+). NAD is a common mediators of various biological processes, including energy metabolism, mitochondrial functions, calcium homeostasis, antioxidation/generation of oxidative stress (8). During cerebral ischemia, NAD is rapidly depleted, and increasing NAD has been proposed as a potential therapeutic strategy against stroke (9). Both NADPH and NAD+ have been reported to display potent neuroprotective effects in ischemia-related neuronal injury (10).

After an ischemic stroke, a series of inflammatory events takes place, leading to activation of resident microglia and mobilization of peripheral leukocytes with their subsequent infiltration into the injured side (11). Infiltrating leukocytes release inflammatory mediators, amplifying the intrinsic brain inflammatory response. Theoretically, these mechanisms could lead to activation of the KP, mainly its “neurotoxic” branch, contributing to neuronal damage and, ultimately, to clinical outcome (3). There is some pre-clinical and clinical evidence indicating activation of KP in stroke. Besides playing a pathophysiological role, KP metabolites have been tested as pharmacological agents to prevent and/or minimize stroke-related brain damage.

In this systematic review, our main objective was to summarize the evidence on the involvement of KP in both experimental models of brain ischemia and human patients with stroke.

## Methods

### Systematic Search

We undertook a comprehensive systematic search to identify all published studies evaluating KP in clinical studies and animal models of brain ischemia or stroke using the electronic databases PubMed, Scopus, and Embase. We conducted the search based on the keywords: (“kynurenine” or “kynurenic acid” or “quinolinic acid”) AND (“ischemia” or “stroke” or “occlusion”). Studies published through November 2018 were included.

**Figure 2:**
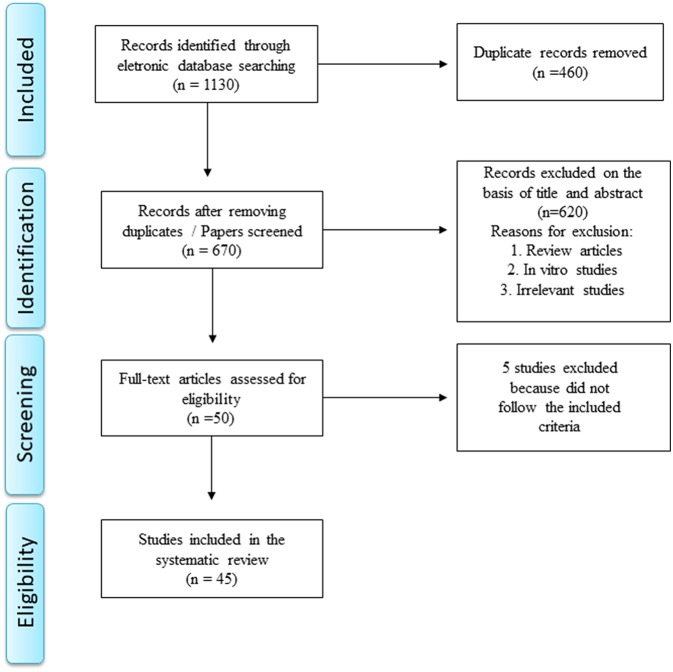
Flow diagram.

A total of 1,130 papers was retrieved after removing duplicate manuscripts. Reference management software (EndNote X7 for Windows from Thomson Reuters, 2013) was used for screening purposes. The systematic review was performed in accordance with the Preferred Reporting Items for Systematic Reviews and Meta-Analysis (PRISMA) statement (12) as depicted in Figure 2.

### Eligibility Criteria, Data Extraction, and Quality Assessment

Studies were considered eligible to be included in the review if they met the following criteria: (1) animal model of stroke or brain ischemia and evaluation of KP metabolites or enzymes; (2) study with stroke patients and evaluation of KP metabolites or enzymes; (3) available in English; and (4) original data (not a review). The exclusion criteria were: (1) review manuscripts, (2) *in vitro* studies, (3) only abstracts and (4) non-relevant studies. During December 2018 and January 2019, one author (GDC) screened the titles and abstracts of all articles. Full texts were obtained for all articles that met the inclusion criteria (*n* = 50). Full-texts of the eligible studies were independently screened by two authors (GDC and ALT) who identified aims, methods, results and conclusions, extracting the respective information. Five papers were excluded in this phase.

To assess the risk of bias of the pre-clinical studies included in this systematic review, we used the SYRCLE's RoB tool (13). This tool evaluates specific features that might be source of bias in animal studies such as group allocation, if animals were randomly housed during the experiment, blinded investigators, animals selected at random for outcome assessment.

## Results

In the end of the screening process, 45 studies met the inclusion criteria and they were included in this systematic review. Among them, 39 studies involved animal models of stroke/ brain ischemia and six were clinical studies comprising patients with stroke. These studies are summarized in Tables 1, 2, while the main results are summarized in Table 3.

**Table 1:** Animal model studies with stroke/ischemia and kynurenine pathway.

**References**	**Animal model**	**Results**	**Conclusion**
Simon et al. (14)	Kynurenate in anoxic ischemic brain injury in neonatal rats	These results show a marked attenuation in brain water in ischemic hemispheres of kynurenate-treated 7-day-old animals that is greatest at 24 h. In regard to neuronal proliferation and migration, the gestational age of 7 days in rats is compatible with that of newborn humans.	The results suggest that kynurenate treatment attenuated brain edema immediately after and 24 h following anoxia-ischemia.
Germano et al. (15)	KYNA in focal cerebral ischemia in adult male Sprague-Dawley rats.	Pre-ischemia but not 1 h post-ischemia treatment with kynurenate attenuated infarction size and improved neurological outcome at 24 h after injury.	The results suggest the role of excitatory neurotransmission in acute neuronal injury and supports pharmacological inhibition of cell excitation as a potential therapy for stroke.
Andine et al. (16)	KYNA in hypoxic-ischemia in neonatal rats.	KYNA reduced weight of lesioned hemisphere.	The results suggest that excitatory amino acids are involved in the development of post ischemic damage in the immature brain.
Roussel et al. (17)	Kynurenate in male Wistar-Kyoto (spontaneously hypertensive) SHRs rats with cerebral occlusion	Kynurenate did not significantly modify either the infarct volume, measured 48 h after occlusion, or the neurological score.	The results suggest that the absence of a neuroprotective effect of kynurenate, which contrasts with results in normotensive rats, is possibly due to impaired collateral circulation in spontaneously hypertensive rats.
Heyes and Nowak (18)	Metabolism of the endogenous excitotoxin, QUIN in ischemia in female Mongolian gerbils	High brain TRP levels, and increased 5-hydroxyindoleacetic acid, occurred during the first hours of recirculation, but regional brain QUIN concentrations decrease during the first 24 h after the ischemia. Increases in QUIN concentrations occurred in striatum and hippocampus at 2 days of recirculation after ischemia. After, 4 and 7 days, QUIN increases in all regions.	The results do not support a role for increased QUIN concentrations in early excitotoxicity-dependent neuronal damage. The role of the delayed increases in brain QUIN in the progression of post ischemic injury remains to be established.
Roucher et al. (19)	Kynurenate in ischemia in male Wistar rat.	Kynurenate had no effect on cerebral metabolism before ischemia. During a 30 min ischemia, kynurenate protected against the decrease in phosphocreatine and the increase in phosphate whereas there was no difference in the decrease in intracellular pH and ATP. The recovery of PCr, Pi, and pHi to control levels during recirculation was faster in the treated group than in the reference group, whereas the time course of ATP recovery was similar in both groups.	The results suggest that kynurenate protects against neuronal loss by mechanisms other than metabolic protection.
Katayama et al. (20)	KYNA administered in transient ischemia in gerbils and Sprague-Dawley rats.	KYNA attenuates the increase in extracellular concentration of lactate during ischemia and attenuates death of hippocampal CA1 pyramidal cells after 5-min transient ischemia in gerbils.	The results suggest that KYNA attenuates the death of hippocampal cells after ischemia in gerbils. The protective effect of KYN may be attributable, in part, to inhibition of lactate accumulation.
Lekieffre et al. (21)	KYNA in male Wistar rats with ischemia.	KYNA decreased glutamate release during ischemia but had no effect on the hippocampal lesion. Some protection was observed in the cortex and in the striatum.	The results suggest that the extracellular accumulation of glutamate during forebrain ischemia does not play a major role in hippocampus damage.
Nozaki and Beal (22)	KYNA in hypoxia-ischemia in 7-day-old rats	L-KYN 1 h before the hypoxia-ischemia showed a dose-dependent neuroprotective effect, with complete protection at a dose of 300 mg/kg. The induction of c-fos immunoreactivity in cerebral cortex was also blocked by this dose of L-KYN. Probenecid alone had moderate neuroprotective effects, while a combination of a low dose of probenecid with doses of 50–200 mg kg-1 of L-KYN showed significant dose-dependent neuroprotection. KYN dose-dependently protected against NMDA neurotoxicity in 7-day-old rats.	The results show for the first time that pharmacological manipulation of endogenous concentrations of KYNA exerts neuroprotective effects.
Saito et al. (23)	IDO activity and QUIN concentrations in transient ischemia in the female Mongolian gerbil.	Increases in IDO activity and QUIN concentrations were found 4 days after ischemia, with responses in hippocampus >striatum >cerebral cortex >thalamus. IDO and QUIN concentrations were unchanged in the cerebellum of post ischemic gerbils. Marked increases in the activity of kynureninase, kynurenine 3-hydroxylase, and 3-hydroxyanthranilate-3, 4-dioxygenase were also detected in hippocampus but not in cerebellum on day 4 of recirculation. Accumulation of QUIN was demonstrated in cerebellum and hippocampus of control gerbils following an intracisternal injection of 3-HAA, which verifies the availability of precursor to both regions when administered intracisternally.	The results suggest that kynurenine 3-hydroxylase may have an important role in determining the flux of kynurenine in brain.
Saito et al. (24)	KYN and QUIN in ischemic brain in female Mongolian gerbils and after systemic administration of pokeweed mitogen	One day after ischemia, kynureninase and 3-hydroxy-3,4-dioxygenase were increased in the hippocampus, but local QUIN levels and the activities of the IDO and kynurenine-3-hydroxylase were unchanged. By days 2 and 4 after ischemia, the activities of all these enzymes in the hippocampus as well as QUIN levels were increased. KAT activity in the hippocampus was unchanged on days 1 and 2 after ischemia but was decreased on day 4, at a time when local KYNA levels were unchanged. Systemic administration of pokeweed mitogen increased indoleamine-2,3-dioxygenase and kynureninase activities in the brain without changes in kynurenine-3-hydroxylase or 3-hydroxyanthranilate-3,4-dioxygenase activities.	The results support a role for macrophage infiltration and increased activity and colocalization of IDO, kynurenine-3-hydroxylase and kynureninase in conveying the ability of brain tissue to convert L-tryptophan to QUIN in neuroinflammation.
Saito et al. (25)	3-hydroxylase activity in female Mongolian gerbils and male Sprague-Dawley rats, male macaques and humans.	Gerbil brain activities ranged from 20 to 50 nmol/g/h, while kynurenine 3-hydroxylase activities in other species were one order of magnitude lower. Kynurenine 3-hydroxylase was also detected in lung, kidney, spleen, intestine, and liver of gerbils, with activities larger than in brain.	The results suggest that delayed increase in KYN pathway metabolism occurs in different brain regions following transient ischemia in the gerbils. These changes are marked in regions that show the most extensive damage and inflammatory responses and are consistent with a localization of the induction of KYN pathway to reactive macrophages.
Zoli et al. (26)	Indole-pyruvic acid (IPA) in male Sprague-Dawley rats.	Analysis showed a protective effect of IPA treatment on striatal ischemic lesions. Increased neuronal loss, regional atrophy and glial fibrillary acidic protein immunoreactivity were observed in the hippocampal formation, especially the CA3 field, of IPA-treated rats when compared with vehicle-treated ischemic rats. The treatment with IPA did not produce any improving effects in a test assessing short-term impairments after transient ischemia. No effects of IPA on performance in water T-maze studied at 7 and 14 days post-ischemia.	The results suggest that IPA does not have neuroprotective effects against ischemia in the hippocampus.
Ghribi et al. (27)	KYNA in ischemia in male Wistar rats	KYNA markedly depressed the ischemia-induced increase in glutamate and aspartate concentrations.	The results indicate that during ischemia, local glutamate receptors play a major role in glutamate and aspartate accumulation in the striatum. Ischemia increase in extracellular concentration of excitatory amino acids may be due in part to a positive glutamatergic feedback loop via activation of NMDA and/or non-NMDA receptors.
Saito et al. (28)	3-hydroxy-anthranilate-3,4-dioxygenase and dexamethasone in female Mongolian gerbils with ischemia.	3-hydroxy-anthranilate-3,4-dioxygenase significantly reduced the accumulation of QUIN in all brain regions in response to cerebral ischemia.	The results suggest that increased QUIN concentration in specific brain regions is linked with enhanced activities of IDO and other kynurenine pathway enzymes.
Heyes et al. (29)	Quinolinate responses to systemic and brain immune activation in gerbils, Sprague-Dawley and Wistar rats	Global cerebral ischemia in gerbils, but not rats, increased hippocampus indoleamine-2,3-dioxygenase activity and quinolinate levels 4 days post injury. In rats, small increases in quinolinate concentrations occurred in infarcted regions on days 1, 3, and 7, although concentrations remained below serum values.	The results demonstrate a limited capacity of rats to elevate in brain and blood QUIN levels in response to immune activation is attributable to increases in local indoleamine-2,3-dioxygenase activity and a low capacity of microglia, astrocytes, and macrophages to convert L-tryptophan to quinolinate.
Baratte et al. (30)	QUIN immunoreactivity in adult male mongolian gerbils with transient global ischemia.	Neurodegeneration was evident in hippocampus 4, 7, and 14 days after ischemia. QUIN positive cells, with microglia-like morphology, appeared in the subiculum and in the CA1, 4 days after ischemia. At 7 days post-ischemia they extended to the whole CA1, disappearing at 14 days.	The results suggest that microglia-like cells infiltrating the degenerating areas of the hippocampus represent the major source of QUIN following transient ischemia in the gerbil. Thus, *in situ* production of QUIN in vulnerable brain regions may contribute to the pathophysiological mechanisms of delayed brain injury.
Cozzi et al. (31)	mNBA and Ro 61-8048 in Mongolian gerbils and male Sprague-Dawley rats.	The percentage of lesioned pyramidal neurones decreased in gerbils. Also, significant reduction in infarct volumes in rats with the inhibition of kynurenine hydroxylase.	The results suggest that inhibition of kynurenine hydroxylase could be a new avenue to reduce neuronal loss in brain ischemia.
Phillis et al. (32)	KYNA in ischemic cerebral cortex in Male Sprague–Dawley rats.	KYNA, administered topically onto the cortex in artificial cerebrospinal fluid using bilateral cortical cups, failed to attenuate ischemia-evoked release of aspartate, glutamate, phosphoethanolamine, taurine and at 1 mM it depressed GABA release.	The results suggest that ischemia-evoked amino acid release is not a significant consequence of the activation of ionotropic receptors by glutamate.
Salvati et al. (33)	KYNA (400–1,600 mg/kg) or vehicle were administered i.p. 15 min before 5 min bilateral carotid occlusion in adult male Mongolian gerbils.	Seven days after reperfusion, ischemia-induced hippocampal nerve cell loss was lower in KYNA-treated gerbils. Treatment with 1,000 mg/kg produced brain KYNA concentrations that were dramatically elevated, as measured in a separate group of transcardially-perfused gerbils. Cerebral KYNA concentrations tended to return to basal values 2 h after reperfusion.	The results indicate that KYNA has a neuroprotective effect in a model of forebrain ischemia, compatible with *in vitro* affinity of this molecule for ionotropic glutamate receptors.
Edwards et al. (34)	Concentrations of glutamate and kynurenate after diclofenac or noxious stimulation in Male Sprague Dawley rats	The glutamate concentrations were lower in the ischemia group when compared with control and diclofenac-treated groups. Diclofenac-treated groups found significant increases in kynurenate concentrations in the diencephalon and lumbo-sacral regions of the CNS.	The results suggest that noxious stimulation from tail ischemia appeared to be associated with increased release of glutamate. NSAIDs increase kynurenate concentrations in the spinal cord and diencephalon. Antagonism by kynurenate of glutamate effects at NMDA receptors may contribute to the antinociceptive effects of NSAIDs
Moroni et al. (35)	Kynurenine hydroxylase inhibitors in gerbils, rats and organotypic rat culture.	In organotypic hippocampal slice cultures deprived of oxygen and glucose, these inhibitors reduced neuronal damage. In gerbils the administration of mNBA or Ro 61-8048 decreased the percentage of damaged pyramidal neurons in the hippocampal CA1 region. In rats Ro 61-8048 administration reduced the infarct volume.	The results demonstrate that ischemic neuronal damage can be decreased by inhibiting kynurenine hydroxylase.
Luchowska et al. (36)	Levels of KYNA after global ischemia in Mongolian gerbils	The level of KYNA in CA1 area was not altered 24 and 72 h following transient global. Similarly, the activities of KATs in CA1 area were not changed.	The results indicate that KYNA production is preserved in CA1 area of gerbil hippocampus during early stages after ischemic insult.
Moroni et al. (37)	KMO in gerbils and organotypic rat hippocampal slice	In gerbils, completely prevented the increase in Glu output induced by transient occlusion of the carotids. In rat hippocampal slices exposed for 30 min to OGD, KMO inhibitors reduced post-ischemic neuronal death and increased KYNA concentrations in the incubation medium. On the contrary, 3-HK added to slices exposed to OGD in the presence of KMO inhibitors completely prevented the neuroprotective effects of the inhibitors.	The results suggest that KMO inhibitors prevent neuronal death by decreasing 3-HK synthesis.
Sas et al. (38)	L-KYN effects in New Zealand white rabbits with ischemic.	L-KYN produced an increase in the normal cCBF. The cCBF-improving effect of L-KYN was immediate and highly in rabbits with carotid occlusion. Pretreatment with atropine or Nomega-nitro-L-arginine-methyl-ester (L-NAME) prevented the L-KYN induced enhancement of the normal and the ischemic cCBF alike.	The results suggest that the cCBF increasing effect of L-KYN might be mediated by activation of cholinergic and nitric oxide pathways.
Abo et al. (39)	KYNA in female Fischer rats	KYNA improved behavioral recovery within 10 days from paralysis induced by the focal PIT, as evaluated with beam walking.	The results suggest that intrathecal administration of a glycine receptor antagonist may facilitate behavioral recovery during the acute phase after brain infarction.
Gigler et al. (40)	MCAO in male NMRI mice + BCCAO in gerbils	Pretreatment with systemic administration of KYN (i.p.) reduced infarct size (in mice) and hippocampal CA1 pyramidal cell loss, preventing post-stroke behavioral changes (hypermotility and decreased spontaneous alternation) in gerbils.	The results suggest that L-KYN can increase the brain concentration of KYNA to neuroprotective levels, suggesting the potential clinical usefulness of L-KYN for the prevention of neuronal loss.
Robotka et al. (41)	L-KYN in ischemic in adult Wistar rats	L-KYN administration decreased the number of neurons injured in the cortex, not only in the pre-treated animals, but also in those which received L-KYN after the ischemic insult.	The results show that even the post-event administration of L-KYN may be of therapeutic benefit in the treatment of global brain ischemia.
Sas et al. (42)	t4VO in adult male Wistar rats	The systemic administration (i.p.) of KYN + probenecid (only pre-treatment strategy) reduced hippocampal CA1 pyramidal cell loss and preserved LTP expression at the Schaffer collateral-CA1 synapses.	The results suggest that administration of KYN elevates KYNA concentration in the brain to neuroprotective levels, suggesting its potential clinical usefulness for the prevention of neuronal loss in neurodegenerative diseases.
Hoshi et al. (43)	tBCCAO in adult male C57BL/6J mice	IDO is overexpressed in hippocampal CA1 area 72 h post-ischemia and is co-localized with Neu-N. This upregulation is possibly independent of IFN-γ.	The results show that up-regulation of IDO in hippocampal neurons after transient global ischemia occurs via INF-y-independent mechanisms.
Sas (44)	KYN in adult male Wistar rats with ischemia and L-KYN and New-Zealand white rabbits with ischemic condition	L-KYN + Probenecid cause neuroprotection in a global ischemic rat model. L-KYN increase the blood flow in control rabbits and ischemic animals.	The results demonstrate that KYN treatment minimizes neuronal cell loss in a rat model of global ischemia in which excitotoxicity seems to play a major pathophysiological role.
Gellert et al. (45)	4VO in adult male Wistar rats	The systemically administered KYNA analog (both pre and post-treatment strategies) reduced hippocampal CA1 pyramidal cell loss and preserved LTP expression at the Schaffer collateral-CA1 synapses.	The results suggest that the neuroprotective effect was robust in the pretreatment, and at the time of reperfusion.
Jackman et al. (46)	tMCAO in adult male C57BL/6 mice	IDO1 is overexpressed (immunohistochemistry) in cerebral arterioles 24 h post-ischemia, with increased KYN/TRP ratio. After stroke, IDO^−/−^ and 1-MT-treated animals had similar pathological and neurological scores than WT animals.	The results suggest that the expression and the activity of IDO1 increase following stroke. However, such IDO1 increased expression does not appear to affect overall outcome following acute ischemic stroke.
Hsieh et al. (47)	KYNA in heatstroke in adult male Sprague-Dawley rats	KYNA decreased the survival time. Vehicle-treated heatstroke rats displayed hypotension, hypothalamic neuronal degeneration and apoptosis, increased serum levels of TNF-α, ICAM-1, and IL-10, and spleen, liver, kidney, and lung apoptosis. KYNA preconditioning protected against hypotension but not hyperthermia and attenuated hypothalamic neuronal degeneration and apoptosis during heatstroke. KYNA preconditioning attenuated spleen, kidney, liver, and lung apoptosis and up-regulated serum IL-10 levels but down-regulated serum TNF-α and ICAM-1 levels during heatstroke.	The results suggest that systemic delivery of KYNA may attenuate multi-organ dysfunction in rats after heatstroke.
Gellert et al. (48)	MCAO in adult male Wistar rats.	The systemically administered L-KYN sulfate (after reperfusion) worsened neuronal loss and glial reaction in the somatosensory cortex.	The results suggest that treatment with L-KYNs worsened the histopathological outcome of dMCAO. This contradictory result indicates that post-ischemic treatment with L-KYNs may be harmful.
Cuartero et al. (49)	MCAO in adult male C57BL/6 mice + oxygen- glucose deprivation in rat cortical neurons	AhR is overexpressed 5 to 72 h post-ischemia. AhR^−/−^ or animals treated with AhR antagonists had decreased infarct size and neurological deficits. The administered L-KYN increased infarct size in an AhR-dependent manner. *In vitro* results confirmed that KYN is a specific AhR agonist in neurons. TDO (but not IDO1 or IDO2) is overexpressed 5 to 24 h post-ischemia and is co-localized with Neu-N. TDO inhibitor (680C91) reduced infarct size, but not IDO1 inhibitor (1-MT).	The results suggest that a L-KYN/AhR pathway mediates acute brain damage after stroke, opening new possibilities for the diagnosis and treatment.
Lee et al. (50)	Protein levels of KYNA after ischemic preconditioning in male Mongolian gerbils	In the ischemia-operated group, a loss of pyramidal neurons in the CA1 stratum pyramidale (SP) at 5 days post-ischemia; however, in the IPC + ischemia-operated group, the pyramidal neurons were protected. KYNA immunoreactivity in the SP of the ischemia-operated group was altered following ischemia-reperfusion and was very low 5 days following ischemia-reperfusion. In the IPC + ischemia-operated group, however, KYNA immunoreactivity was detected in the SP of the CA1 region after the ischemic insult. Alteration of the KYNA protein level in the CA1 region following ischemia was similar to the immunohistochemical changes observed.	The results indicate that the enhancement of KYNA expression by IPC may be necessary for neuronal survival following transient ischemic injury.
Mangas et al. (51)	tMCAO in adult male Wistar rats	KYNA is overexpressed (immunohistochemistry) in infarcted areas from day 2 to 21 post-ischemia and is co-localized with GFAP.	The results suggest that KYNA could be involved in neuroprotective, scavenger, and/or antioxidant mechanisms.
Mangas et al. (52)	New therapeutic approach Gemst in stroke in male Wistar rat brain.	Gemst reversed the pathological conditions of stroke to normal situations. Gemst exerts a multifunctional action: down-regulates the indoleamine 2, 3-dioxygenase pathway and abolishes brain infiltration, microglial activation and gliosis. Moreover, Gemst has no effect on the expression of doublecortin, a protein involved in neuronal migration.	The results suggest that Gemst could be a new drug for the treatment of stroke since it reverses the pathological findings of stroke and normalizes brain tissue conditions following an ischemic insult.

**Table 2:** Humans studies with stroke/ischemia and kynurenine pathway.

**References**	**Study population**	**Results**	**Conclusion**
Darlington et al. (53)	Patients with symptoms of acute stroke. Performed CY scan and measure kynurenines, neopterin, lipid peroxidation, S100B	KYN pathway of tryptophan metabolism is activated, with an increased KYN:TRP ratio, but with a highly significant decrease in the ratio of 3-HAA: anthranilic acid, which was strongly correlated with infarct volume. Levels of KYNA were significantly raised in patients who died within 21 days compared with those who survived. The results suggest that increased TRP catabolism is initiated before or immediately after a stroke, and is related to the inflammatory response and oxidative stress, with a major change in 3-HAA levels.	The results suggest that oxidative tryptophan metabolism may contribute to the oxidative stress and brain damage following stroke. Some form of anti-inflammatory intervention between the rise of S100B and the activation of microglia, including inhibition of the kynurenine pathway, may be valuable in modifying patient morbidity and mortality
Brouns et al. (54)	Plasma concentrations of TRP and its metabolites were measured in 149 stroke patients at admission, at 24 h, at 72 h and at day 7 after stroke onset.	KYN/TRP but not KA/3-HAA correlated with the NIHSS score and with the infarct volume. Patients with poor outcome had higher mean KYN/TRP ratios than patients with more favorable outcome. The KYN/TRP ratio at admission correlated with CRP levels, ESR and NLR. The activity of the kynurenine pathway for tryptophan degradation in acute ischemic stroke correlates with stroke severity and long-term stroke outcome.	The results suggest the activity of the KYN pathway for tryptophan degradation in acute ischemic stroke correlates with stroke severity and long-term stroke outcome. Tryptophan oxidation is related to the stroke induced inflammatory response.
Gold et al. (55)	Patients were recruited from the acute stroke. Assessments for cognition, stroke severity, and the depressive symptoms. TRP and KYN concentrations were determined by high-performance liquid chromatography.	Higher KYN/TRP ratios were associated with lower post-stroke global cognition. A backward stepwise elimination linear regression showed that the highest KYN/TRP ratio tertile predicted lower sMMSE scores, controlling for age with NIHSS, and lesion volume.	The results suggest an inflammatory response characterized by IDO activation may be relevant to the development of PSCI. Since the neuroactivity of KYN metabolites may be amenable to pharmacotherapeutic intervention, the KYN/TRP ratio may be a clinically important biomarker.
Bensimon et al. (56)	Patients with ischemic stroke divided into high, medium, and low depressive symptom tertiles. Measure concentrations of KYN and TRP and cytokine concentrations.	No differences in KYN/TRP ratios between CES-D for cytokines (*n* = 53), serum IL-1β concentrations and serum ratios of IL-18/IL-10, IFNγ/IL-10, and IL-1β/IL-10 were elevated in the middle CES-D tertile. *Post hoc* analyses suggested that serum ratios of IL-18/IL-10, and IL-1β/IL-10, as well as IL-1β, were significantly associated with fatigue.	The results suggest that peripheral KYN/TRP ratios were not associated with depressive symptoms in a post-stroke population. However, in exploratory analyses a pro-inflammatory bias was identified specifically in patients with mild depressive symptoms and associated with post-stroke fatigue, suggesting an avenue for future research.
Mo et al. (57)	A total of 81 patients with ischemic stroke and 35 normal controls were recruited. Measure, concentration of serum hsCRP, apolipoprotein A-1 and apolipoprotein B, triglyceride, cholesterol, high density lipoprotein (HDL). TRP, KYN and KYNA.	Lower TRP, KYNA, HDL, and KAT activity ratio were found in the stroke group compared to the control group. The levels of hsCRP and IDO activity ratio were much higher in the stroke group than the control group. The IDO activity in patients with ischemic stroke showed a positive correlation with hsCRP. In addition, hsCRP and IDO levels were positively associated with the NIHSS score both at admission and 3 weeks post-stroke.	The results suggest an inflammatory response characterized by up-regulated IDO activation in ischemic stroke, which might be closely relevant to its pathophysiology.
Ormstad et al. (58)	Acute serum levels of 5-hydroxytryptamine (5-HT), TRP catabolites (TRYCATs), and competing amino acids, as well as subsequent fatigue and depression, were measured in 45 stroke patients.	TRP index [ = 100 × TRP / (tyrosine + valine + phenylalanine + leucine + isoleucine)] was lower in patients with a Fatigue Severity Scale (FSS) score of ≥4 at 12 months than in those with an FSS score of <4. Furthermore, the serum level of KYNA in the acute stroke phase was higher in patients with an FSS of score ≥4 at 18 months than in those with an FSS score of <4. These findings indicate that stroke patients with PSF have a lower bioavailability of TRP for 5-HT synthesis in the brain in the acute stroke phase.	The findings indicate that stroke patients with PSF have a lower bioavailability of TRP for 5-HT synthesis in the brain in the acute stroke phase. However, they also appear to have greater neuroprotective potential in that phase. In contrast to PSF, no predictors of PSD were found.

**Table 3:** Main results of KYN pathway and stroke.

**Specie**	**KYN**		**KYNA**		**QA**		**IDO**		**TRP**		**Treatments**	
	**Periphery**	**Brain**	**Periphery**	**Brain**	**Periphery**	**Brain**	**Periphery**	**Brain**	**Periphery**	**Brain**	**KYNA/KYN/analog**	**IDO inhibitors**
Rats	NA	NA	NA	↑	NA	↑	NA	NA	NA	NA	Protection/Worsened/ no effect	Protection
Gerbils	NA	NA	NA	No changes	NA	↑/↓	NA	↑	NA	NA	Protection	Protection
Mice	NA	NA	NA	NA	NA	NA	NA	↑	NA	NA	Protection	NA
Rabbit	NA	NA	NA	NA	NA	NA	NA	NA	NA	NA	Protection	NA
Humans	↑	NA	↓/↑	NA	NA	NA	↑	NA	↓	NA	NA	NA

↓ levels decreased; ↑ levels increased.

### Pre-clinical Studies

Regarding the quality assessment of the pre-clinical studies, only five papers reported that randomization was carried out, but without stating the method of group allocation. All studies reported that age and/or weight were similar among groups. None of the studies described whether animals were randomly selected for outcome assessment.

The type of animal model varied among the studies. More than 25 studies used rats while the second most used animal was the Mongolian gerbil followed by mice. Two studies used rabbits. Only six studies used females: one study with rats and five studies with gerbils, but none specifically examined the effects by sex. No study assessed aged animals.

The main aim of pre-clinical investigation was to assess the neuroprotective effects of KP metabolites (kynurenine or kynurenic acid) and/or KMO inhibitors (mNBA and Ro 61-8048) in different models of ischemia. Overall, studies with kynurenine and KMO inhibitors showed positive results, i.e., decrease in the infarct size and increased survival with treatment.

Gigler et al. (40) showed that intraperitoneal (i.p.) administration of kynurenine before stroke by middle cerebral artery occlusion reduced infarct size and hippocampal CA1 pyramidal cell loss in adult male mice. This same strategy also prevented post-stroke behavioral changes in gerbils in the bilateral carotid occlusion model (40). Pre- and post-treatment with kynurenine decreased cortical neuron damage in adult rats subjected to four-vessel occlusion (41).

Cozzi et al. evaluated the neuroprotective effects of KMO inhibitors. The KMO inhibitors mNBA and Ro 61-8048 were tested as neuroprotective strategies against ischemia induced by bilateral carotid occlusion in male Mongolian gerbils or middle cerebral artery occlusion in male rats. In gerbils, the percentage of lesioned pyramidal neurons in the hippocampal CAl region decreased significantly from around 90% in vehicle-treated animals to 7% after mNBA (400 mg/kg i.p., three times at 1, 30, and 180 min after occlusion) or 10% after Ro61-8048 (40 mg/kg i.p., three times at 1, 30, and 360 min after occlusion). A major reduction in infarct volumes was also found in rats after middle cerebral artery occlusion treated with mNBA or Ro 61-8048 administrated 30 min after the occlusion (31).

The results are mixed regarding the effects of kynurenic acid and analogs. Kynurenic acid did not show any protective effects in two studies (17, 48). Roussel et al. for instance, showed that kynurenic acid did not modify neither infarct volume or the neurological score 48 h after middle cerebral artery occlusion in adult male rats (17). Conversely, Gellert et al. (45) showed that administration of a kynurenic acid analog (2-(2-N,N-dimethylaminoethylamine-1-carbonyl)-1H-quinolin-4-one hydrochloride) either before or after ischemia reduced hippocampal CA1 pyramidal cell loss and preserved long-term potentiation expression at the Schaffer collateral-CA1 synapses in adult male rats in a four-vessel occlusion model. Other study showed that pretreatment with systemic administration of KYN (i.p.) reduced infarct size in mice and hippocampal CA1 pyramidal cell loss in gerbils, preventing post-stroke behavioral changes as hypermotility and decreased spontaneous alternation (40).

Pre-clinical studies also addressed the pathophysiological involvement of the KP after stroke, mainly focusing on the expression of the enzyme IDO and the metabolite kynurenic acid. Hoshi et al. showed by mRNA and immunohistochemistry increased expression of IDO in hippocampal CA1 area of adult male mice 72 h after global cerebral ischemia and that IDO is co-localized with Neu-N, a neuronal marker (43). Increased IDO expression was also observed in endothelial cells from cerebral arterioles 24 h post-middle cerebral artery occlusion, with increased plasma kynurenine/tryptophan ratio, an index of IDO activity, in adult male mice (46). Increased IDO activity and quinolinic acid concentrations in hippocampus, striatum, cortex, and thalamus, but not in cerebellum, were observed 4 days after 10 min of ischemia by bilateral common carotid artery occlusion in female Mongolian gerbils. Increased activity of kynureninase, kynurenine 3-hydroxylase, and 3-hydroxyanthranilate-3, 4-dioxygenase was also observed in hippocampus but not in cerebellum on day 4 (23). Most studies found increased expression of kynurenic acid in the stroke area. Kynurenic acid expression was increased in the infarcted areas from day 2 to 21 post-ischemia caused by single transient middle cerebral artery occlusion in adult male rats (52).

### Clinical Studies

Only six clinical studies were identified. In these studies, patients were recruited within the first hours of the stroke, and different molecules such as tryptophan, kynurenine and kynurenic acid were measured in parallel with clinical parameters. Only two studies compared stroke patients with healthy controls, while the remaining four investigated whether KP-related molecules could be used as biomarkers of prognosis or clinical outcomes.

Ximing et al. studied 81 patients with ischemic stroke and 35 healthy controls, and reported a decrease in serum levels of tryptophan, kynurenic acid and KAT activity (calculated by kynurenic acid/kynurenine ratio) in the stroke group. Conversely, the levels of highly sensitive C reactive protein (hsCRP), a marker of inflammation, and IDO activity, as assessed by the kynurenine/tryptophan ratio, were significantly higher in the stroke group compared to controls. IDO activity showed a positive correlation with hsCRP, while hsCRP levels and IDO activity were positively correlated with the severity of the stroke as estimated by the NIH Stroke Scale both at admission and 3 weeks post-stroke (57). The other study investigated whether the KP activation correlated with infarct volume in 50 patients with acute stroke and 35 healthy control subjects (53). Tryptophan levels were significantly lower in stroke patients compared to controls at three time points: 1, 7, and 14 days after stroke. Kynurenine levels were increased in patients compared with controls on the first day post-stroke but not at subsequent time points. These results suggest that increased tryptophan catabolism is initiated immediately after the ischemic event, likely triggered by the inflammatory response and oxidative stress, with a major change in 3-hydroxyanthranilic acid levels that strongly correlated with infarct volume (53). Of note, 3-hydroxyanthranilic acid can generate reactive oxygen species, such as hydrogen peroxide and superoxide, in the presence of transition metal ions (59, 60).

Plasma levels of tryptophan and KP metabolites were measured in 149 stroke patients at admission, 24 h, 72 h, and day 7 after stroke. Patients with poor outcome had higher kynurenine/tryptophan ratio, an index of IDO activity and KP activation, compared to patients with favorable outcomes. Moreover, the activity of IDO, as estimated by kynurenine/tryptophan ratio, correlated with stroke severity and clinical outcome (54). In line with this result, Gold et al. reported higher kynurenine/tryptophan ratio in patients with impaired post-stroke cognition as assessed by the Mini-Mental State Examination within 1 month post-stroke (55).

In the study by Bensimon et al. patients with ischemic stroke were categorized based on their depressive symptoms into high, medium, and low severity in the acute phase of the stroke. Kynurenine and tryptophan were also determined. There was no difference in kynurenine/tryptophan ratio among depressive groups (56).

## Discussion

There is evidence implicating the KP in the pathophysiology of stroke. Preclinical evidence also suggests that treatment with kynurenine and KMO inhibitors can be neuroprotective strategies to decrease infarct size and the related clinical outcomes.

In the KP, tryptophan is degraded to kynurenine that can be converted to kynurenic acid by KATs expressed in astrocytes (3). Kynurenic acid plays neuroprotective roles in the central nervous system as a NMDA and α7-nicotinic acetylcholine receptor antagonist (61). Corroborating this concept, a study with intrathecal administration of kynurenic acid improved motor outcome in rats subjected to photochemically induced brain thrombosis (39). Nevertheless, peripheral administration of kynurenic acid or its agonists did not lead to similar results because its penetration through the blood-brain barrier is poor (62). Interestingly, peripheral administration of kynurenine—that can cross the blood-brain barrier and then be converted to kynurenic acid in astrocytes—improved neurological outcome after stroke (40, 63, 64). In contrast to these reports, Gellert et al. (48) showed that post-ischemic treatment of adult male rats subjected to distal middle cerebral artery occlusion for 30 min with kynurenine resulted in worsened glial reaction and neuronal death (48). The extension of brain damage in the different models and the timing of treatment could explain this contradicting finding as administered kynurenine can be converted to quinolinic acid by KMO expressed in activated microglia and infiltrating leukocytes in the infarct area (6). Quinolinic acid is a neurotoxic agent, acting through multiple mechanisms including NMDA receptor agonism (65). Supporting these assumptions, there is evidence from human studies showing increased levels of quinolinic acid in microglia from the brain of patients with depression (66). Interestingly enough, depressive disorders are very common post-stroke complications, affecting over 30% of patients, which corroborates the view that KP may play a role in the pathophysiology of stroke (67).

Evidence from experimental models of stroke, as presented here, neurodegenerative diseases and other neuropsychiatric conditions such as Alzheimer's disease, Huntington's disease and depression, shows that KMO inhibition prevents neuronal loss and the related cognitive and behavioral changes (5, 7). As the available KMO inhibitors do not cross the blood-brain barrier and KMO is highly expressed in peripheral immune cells such as macrophages, it has been proposed that their peripheral action is responsible for these neuroprotective effects (68). In support of this, peripheral inhibition of KMO leads to sustained elevation in the brain levels of kynurenic acid without increasing quinolinic acid levels in the blood or brain (5). However, increased levels of kynurenic acid in the brain may not always be neuroprotective. Kindler et al. showed that patients with schizophrenia had increased kynurenic acid levels that were associated with brain volume loss and attention impairment (69). Accordingly, treatment in stroke should be aimed at “normalizing” and/or rebalancing the levels of kynurenic acid.

IDO is mainly expressed in monocytes, macrophages, dendritic cells and microglia, the tissue-resident phagocytes of the brain (70). IDO is the rate-limiting enzyme of the KP in these cells, and can be upregulated by pro-inflammatory stimuli, especially interferon-gamma (IFN-γ) (71). Accordingly, IDO is highly expressed in infarcted brain areas. Despite that, only one study so far investigated the potential benefits of IDO inhibitors in stroke or ischemia model. The study from Jackman et al. showed that although IDO expression and activity are increased after transient MCAO in mice, genetic and pharmacological methods for IDO inhibition after ischemia (IDO knockout mice and 1-MT treatment, respectively) had no positive effects on infarct volume and neurological outcome, however the authors only examined the effect of IDO1 expression on outcome at 24 h and no later time points (46). IDO inhibitors could be a potential treatment for stroke, however more studies using IDO inhibitors in stroke are warranted to better understand of its mechanism. It is worth mentioning that inhibitors of IDO1 has being used in experiments in oncology (72). As IDO1 has the ability to establish a tumor-promoting inflammatory environment, IDO1 inhibitors decrease tumor-related inflammation and angiogenesis, potentiating the efficacy of cytotoxic or targeted chemotherapies, radiotherapy, immune checkpoint therapies and cancer vaccines (72). This strategy of decreasing the inflammation by IDO inhibitors could be explored in the treatment of stroke and related complications.

Even though several studies were concordant with the concept that KP-based treatments are potentially neuroprotective in stroke, several methodological limitations must be acknowledged. In pre-clinical studies, there were significant differences in the stroke models (focal vs. global ischemia; permanent vs. transient ischemia), in the species used (rats, Gerbils, mice, rabbits) and in the time/dose of pharmacological manipulations. Quality assessment of these studies also suggested a high risk of bias. Moreover, most studies did not take into account sex differences that have been recognized as relevant in the pathophysiology of stroke (73), while no study evaluated aged animals. In addition, there is only one study investigating KP in endothelial cells. Endothelial cells play a critical role in vascular homeostasis and stroke development, with damaged endothelial cells potentially triggering a series of cerebrovascular injuries (74). Finally, most pre-clinical studies only addressed neuroprotection as assessed by infarct size, with limited evaluation of behavioral measures or underlying mechanisms. More carefully designed studies controlling for age and sex differences, and assessing the cells expressing the KP enzymes are definitely warranted.

Human studies corroborate the experimental findings of KP activation after stroke. Interestingly enough, KP-related biomarkers correlated with stroke severity and clinical outcomes, including cognitive impairment. These findings must be seen as preliminary as most studies did not include a control group and did not perform a careful behavioral, cognitive and socio-occupational phenotyping of the patients. In this regard, it would be important to specifically address which clinical parameters (i.e., neurological impairment, cognitive dysfunction, depression) are better predicted by these biomarkers. Another limitation here would be the standard method for KP metabolites assessment, i.e., liquid chromatography–mass spectrometry (LC-MS) that prevents the scaling of their measurement. In addition, only two studies compared patients with health controls.

In conclusion, KP is a promising target for the development of neuroprotective strategies against stroke.

## Data Availability

All datasets analyzed for this study are included in the manuscript and/or the supplementary material.

## Author Contributions

GC performed the search in the literature, screening of the papers and wrote the manuscript. AT helped screening of the papers, wrote the manuscript and mentored. VV and LM reviewed the manuscript.

### Conflict of Interest Statement

The authors declare that the research was conducted in the absence of any commercial or financial relationships that could be construed as a potential conflict of interest.

## Abbreviations

3-HK: 3-hydroxykynurenine
AhR: aryl hydrocarbon receptor
BCCAO: Bilateral Common Carotid Artery Occlusion
Ccbf: Corticocerebral blood flow
CNS: Central nervous system
GFAP: Glial fibrillary acidic protein
ICAM-1: Intercellular Adhesion Molecule 1
IDO1: indoleamine 2,3-dioxygenase 1
IDO2: indoleamine 2,3-dioxygenase 2
IFN-γ: Interferon gamma
IL-10: interleukin-10
IPC: Ischemia preconditioning
KATs: Kynurenine aminotransferase
KMO: Kynurenine 3-monooxygenase
KYNA: Kynurenic acid
L-KYN: Kynurenine
LTP: Long-term potentiation
MCAO: Middle Cerebral Artery Occlusion
NMDA: N-methyl-D-aspartate
OGD: Glucose deprivation
PIT: Photochemically induced thrombosis
QUIN: Quinolinic acid
4VO: Four-vessel occlusion
TDO: Tryptophan-2,3-dioxygenase
TNF-α: Tumor Necrosis Factor Alpha
TRP: Tryptophan
WB: Western blot.

## References

[1] Hankey GJ. Stroke. Lancet. (2017) 389:641–54. 10.1016/S0140-6736(16)30962-X 27637676

[2] Schwarcz R Bruno JP Muchowski PJ Wu HQ. Kynurenines in the mammalian brain: when physiology meets pathology. Nat Rev Neurosci. (2012) 13:465–77. 10.1038/nrn3257 22678511 PMC3681811

[3] Badawy AA. Kynurenine pathway of tryptophan metabolism: regulatory and functional aspects. Int J Tryptophan Res. (2017) 10:1178646917691938. 10.1177/1178646917691938 28469468 PMC5398323

[4] Kanai M Nakamura T Funakoshi H. Identification and characterization of novel variants of the tryptophan 2,3-dioxygenase gene: differential regulation in the mouse nervous system during development. Neurosci Res. (2009) 64:111–7. 10.1016/j.neures.2009.02.004 19428689

[5] Zwilling D Huang SY Sathyasaikumar KV Notarangelo FM Guidetti P Wu HQ. Kynurenine 3-monooxygenase inhibition in blood ameliorates neurodegeneration. Cell. (2011) 145:863–74. 10.1016/j.cell.2011.05.020 21640374 PMC3118409

[6] Cuartero MI de la Parra J Garcia-Culebras A Ballesteros I Lizasoain I Moro MA. The kynurenine pathway in the acute and chronic phases of cerebral ischemia. Curr Pharm Des. (2016) 22:1060–73. 25248805 10.2174/1381612822666151214125950 PMC4972938

[7] Parrott JM O'Connor JC. Kynurenine 3-monooxygenase: an influential mediator of neuropathology. Front Psychiatry. (2015) 6:116. 10.3389/fpsyt.2015.00116 26347662 PMC4542134

[8] Ying W. NAD+/NADH and NADP+/NADPH in cellular functions and cell death: regulation and biological consequences. Antioxid Redox Signal. (2008) 10:179–206. 10.1089/ars.2007.1672 18020963

[9] Wang P Miao CY. NAMPT as a therapeutic target against stroke. Trends Pharmacol Sci. (2015) 36:891–905. 10.1016/j.tips.2015.08.012 26538317

[10] Huang Q Sun M Li M Zhang D Han F Wu JC. Combination of NAD(+) and NADPH offers greater neuroprotection in ischemic stroke models by relieving metabolic stress. Mol Neurobiol. (2018) 55:6063–75. 10.1007/s12035-017-0809-7 29164394

[11] Ao LY Yan YY Zhou L Li CY Li WT Fang WR. Immune cells after ischemic stroke onset: roles, migration, and target intervention. J Mol Neurosci. (2018) 66:342–55. 10.1007/s12031-018-1173-4 30276612

[12] Moher D Liberati A Tetzlaff J Altman DG Group P Preferred reporting items for systematic reviews and meta-analyses: the PRISMA statement. PLoS Med. (2009) 6:e1000097 10.1371/journal.pmed.1000097 19621072 PMC2707599

[13] Hooijmans CR Rovers MM de Vries RB Leenaars M Ritskes-Hoitinga M Langendam MW. SYRCLE's risk of bias tool for animal studies. BMC Med Res Methodol. (2014) 14:43. 10.1186/1471-2288-14-43 24667063 PMC4230647

[14] Simon RP Young RS Stout S Cheng J. Inhibition of excitatory neurotransmission with kynurenate reduces brain edema in neonatal anoxia. Neurosci Lett. (1986) 71:361–4. 2879266 10.1016/0304-3940(86)90648-8

[15] Germano IM Pitts LH Meldrum BS Bartkowski HM Simon RP. Kynurenate inhibition of cell excitation decreases stroke size and deficits. Ann Neurol. (1987) 22:730–4. 10.1002/ana.410220609 3435082

[16] Andine P Lehmann A Ellren K Wennberg E Kjellmer I Nielsen T. The excitatory amino acid antagonist kynurenic acid administered after hypoxic-ischemia in neonatal rats offers neuroprotection. Neurosci Lett. (1988) 90:208–12. 3412643 10.1016/0304-3940(88)90813-0

[17] Roussel S Pinard E Seylaz J Kynurenate does not reduce infarct size after middle cerebral artery occlusion in spontaneously hypertensive rats. Brain Res. (1990) 518:353–5. 2390725 10.1016/0006-8993(90)90997-p

[18] Heyes MP Nowak TS Jr. Delayed increases in regional brain quinolinic acid follow transient ischemia in the gerbil. J Cereb Blood Flow Metab. (1990) 10:660–7. 10.1038/jcbfm.1990.119 1696582

[19] Roucher P Meric P Correze JL Mispelter J Tiffon B Lhoste JM. Metabolic effects of kynurenate during reversible forebrain ischemia studied by *in vivo* 31P-nuclear magnetic resonance spectroscopy. Brain Res. (1991) 550:54–60. 1889001 10.1016/0006-8993(91)90404-j

[20] Katayama Y Kawamata T Kano T Tsubokawa T. Excitatory amino acid antagonist administered via microdialysis attenuates lactate accumulation during cerebral ischemia and subsequent hippocampal damage. Brain Res. (1992) 584:329–33. 1515951 10.1016/0006-8993(92)90916-w

[21] Lekieffre D Ghribi O Callebert J Allix M Plotkine M Boulu RG Inhibition of glutamate release in rat hippocampus by kynurenic acid does not protect CA1 cells from forebrain ischemia. Brain Res. (1992) 592:333–7. 1360314 10.1016/0006-8993(92)91693-9

[22] Nozaki K Beal MF. Neuroprotective effects of L-kynurenine on hypoxia-ischemia and NMDA lesions in neonatal rats. J Cereb Blood Flow Metab. (1992) 12:400–7. 10.1038/jcbfm.1992.57 1569135

[23] Saito K Nowak TS Jr Markey SP Heyes MP. Mechanism of delayed increases in kynurenine pathway metabolism in damaged brain regions following transient cerebral ischemia. J Neurochem. (1993) 60:180–92. 8417138 10.1111/j.1471-4159.1993.tb05836.x

[24] Saito K Nowak TS Jr Suyama K Quearry BJ Saito M Crowley JS. Kynurenine pathway enzymes in brain: responses to ischemic brain injury versus systemic immune activation. J Neurochem. (1993) 61:2061–70. 8245962 10.1111/j.1471-4159.1993.tb07443.x

[25] Saito K Quearry BJ Saito M Nowak TS Jr Markey SP Heyes MP. Kynurenine 3-hydroxylase in brain: species activity differences and effect of gerbil cerebral ischemia. Arch Biochem Biophys. (1993) 307:104–9. 10.1006/abbi.1993.1567 8239646

[26] Zoli M Merlo Pich E Ferraguti F Biagini G Fuxe K Agnati LF Indole-pyruvic acid treatment reduces damage in striatum but not in hippocampus after transient forebrain ischemia in the rat. Neurochem Int. (1993) 23:139–48. 8369738 10.1016/0197-0186(93)90091-i

[27] Ghribi O Callebert J Plotkine M Boulu RG. Effect of kynurenic acid on the ischaemia-induced accumulation of glutamate in rat striatum. NeuroReport. (1994) 5:435–7. 10.1097/00001756-199401120-00016 7911686

[28] Saito K Seishima M Noma A Suyama K Markey SP Heyes MP. 4-chloro-3-hydroxyanthranilate attenuate quinolinic acid accumulation in brain following transient cerebral ischemia in the gerbil. Adv Exp Med Biol. (1996) 398:407–11. 8906296 10.1007/978-1-4613-0381-7_62

[29] Heyes MP Saito K Chen CY Proescholdt MG Nowak TS Jr Li J. Species heterogeneity between gerbils and rats: quinolinate production by microglia and astrocytes and accumulations in response to ischemic brain injury and systemic immune activation. J Neurochem. (1997) 69:1519–29. 9326281 10.1046/j.1471-4159.1997.69041519.x

[30] Baratte S Molinari A Veneroni O Speciale C Benatti L Salvati P. Temporal and spatial changes of quinolinic acid immunoreactivity in the gerbil hippocampus following transient cerebral ischemia. Brain Res Mol Brain Res. (1998) 59:50–7. 9729272 10.1016/s0169-328x(98)00136-3

[31] Cozzi A Carpenedo R Moroni F Kynurenine hydroxylase inhibitors reduce ischemic brain damage: studies with (m-nitrobenzoyl)-alanine (mNBA) and 3,4-dimethoxy-[-N-4-(nitrophenyl)thiazol-2yl]-benzenesulfonamide (Ro 61-8048) in models of focal or global brain ischemia. J Cereb Blood Flow Metab. (1999) 19:771–7. 10.1097/00004647-199907000-00007 10413032

[32] Phillis JW Song D Guyot LL O'Regan MH. Failure of kynurenic acid to inhibit amino acid release from the ischemic rat cerebral cortex. Neurosci Lett. (1999) 273:21–4. 10505642 10.1016/s0304-3940(99)00612-6

[33] Salvati P Ukmar G Dho L Rosa B Cini M Marconi M. Brain concentrations of kynurenic acid after a systemic neuroprotective dose in the gerbil model of global ischemia. Prog Neuropsychopharmacol Biol Psychiatry. (1999) 23:741–52. 10390731 10.1016/s0278-5846(99)00032-9

[34] Edwards SR Mather LE Lin Y Power I Cousins MJ Glutamate and kynurenate in the rat central nervous system following treatments with tail ischaemia or diclofenac. J Pharm Pharmacol. (2000) 52:59–66. 10.1211/0022357001773698 10716604

[35] Moroni F Cozzi A Peruginelli F Carpenedo R Pellegrini-Giampietro DE. Neuroprotective effects of kynurenine-3-hydroxylase inhibitors in models of brain ischemia. Adv Exp Med Biol. (2000) 467:199–206. 10.1007/978-1-4615-4709-9_26 10721057

[36] Luchowska E Luchowski P Sarnowska A Wielosz M Turski WA Urbanska EM. Endogenous level of kynurenic acid and activities of kynurenine aminotransferases following transient global ischemia in the gerbil hippocampus. Pol J Pharmacol. (2003) 55:443–7. 14506324

[37] Moroni F Carpenedo R Cozzi A Meli E Chiarugi A Pellegrini-Giampietro DE. Studies on the neuroprotective action of kynurenine mono-oxygenase inhibitors in post-ischemic brain damage. Adv Exp Med Biol. (2003) 527:127–36. 10.1007/978-1-4615-0135-0_15 15206725

[38] Sas K Csete K Vecsei L Papp JG. Effect of systemic administration of L-kynurenine on corticocerebral blood flow under normal and ischemic conditions of the brain in conscious rabbits. J Cardiovasc Pharmacol. (2003) 42:403–9. 10.1097/00005344-200309000-00012 12960686

[39] Abo M Yamauchi H Suzuki M Sakuma M Urashima M. Facilitated beam-walking recovery during acute phase by kynurenic acid treatment in a rat model of photochemically induced thrombosis causing focal cerebral ischemia. Neurosignals. (2006) 15:102–10. 10.1159/000094876 16888405

[40] Gigler G Szenasi G Simo A Levay G Harsing LG Jr Sas K. Neuroprotective effect of L-kynurenine sulfate administered before focal cerebral ischemia in mice and global cerebral ischemia in gerbils. Eur J Pharmacol. (2007) 564:116–22. 10.1016/j.ejphar.2007.02.029 17407777

[41] Robotka H Toldi J Vécsei L L-kynurenine: metabolism and mechanism of neuroprotection. Future Neurol. (2008) 3:169–88. 10.2217/14796708.3.2.169

[42] Sas K Robotka H Rózsa É Ágoston M Szénási G Gigler G. Kynurenine diminishes the ischemia-induced histological and electrophysiological deficits in the rat hippocampus. Neurobiol Dis. (2008) 32:302–8. 10.1016/j.nbd.2008.07.013 18761090

[43] Hoshi M Saito K Murakami Y Taguchi A Fujigaki H Tanaka R. Marked increases in hippocampal neuron indoleamine 2, 3–dioxygenase via IFN-gamma-independent pathway following transient global ischemia in mouse. Neurosci Res. (2009) 63:194–8. 10.1016/j.neures.2008.12.003 19121343

[44] Sas K. Potential role of glutamate neurotransmission in the pathogenesis of ischemic brain damage and of depression. Effects of L-kynurenine on the survival of the hippocampal neurons and on the corticocerebral blood flow in ischemic animal models. Ideggyogy Sz. (2010) 63:61–70. 20420126

[45] Gellert L Fuzik J Goblos A Sarkozi K Marosi M Kis Z. Neuroprotection with a new kynurenic acid analog in the four-vessel occlusion model of ischemia. Eur J Pharmacol. (2011) 667:182–7. 10.1016/j.ejphar.2011.05.069 21664350

[46] Jackman KA Brait VH Wang Y Maghzal GJ Ball HJ McKenzie G. Vascular expression, activity and function of indoleamine 2,3-dioxygenase-1 following cerebral ischaemia-reperfusion in mice. Naunyn Schmiedebergs Arch Pharmacol. (2011) 383:471–81. 10.1007/s00210-011-0611-4 21359968

[47] Hsieh YC Chen RF Yeh YS Lin MT Hsieh JH Chen SH. Kynurenic acid attenuates multiorgan dysfunction in rats after heatstroke. Acta Pharmacol Sin. (2011) 32:167–74. 10.1038/aps.2010.191 21293468 PMC4009940

[48] Gellert L Knapp L Nemeth K Heredi J Varga D Olah G. Post-ischemic treatment with L-kynurenine sulfate exacerbates neuronal damage after transient middle cerebral artery occlusion. Neuroscience. (2013) 247:95–101. 10.1016/j.neuroscience.2013.04.063 23685169

[49] Cuartero MI Ballesteros I de la Parra J Harkin AL Abautret-Daly A Sherwin E. L-kynurenine/aryl hydrocarbon receptor pathway mediates brain damage after experimental stroke. Circulation. (2014) 130:2040–51. 10.1161/circulationaha.114.011394 25359166

[50] Lee JC Tae HJ Cho GS Kim IH Ahn JH Park JH. Ischemic preconditioning protects neurons from damage and maintains the immunoreactivity of kynurenic acid in the gerbil hippocampal CA1 region following transient cerebral ischemia. Int J Mol Med. (2015) 35:1537–44. 10.3892/ijmm.2015.2171 25872573 PMC4432926

[51] Mangas A Yajeya J Gonzalez N Ruiz I Duleu S Geffard M. Overexpression of kynurenic acid in stroke: an endogenous neuroprotector? Ann Anat. (2017) 211:33–8. 10.1016/j.aanat.2017.01.002 28163204

[52] Mangas A Yajeya J Gonzalez N Ruiz I Pernia M Geffard M. Gemst: a taylor-made combination that reverts neuroanatomical changes in stroke. Eur J Histochem. (2017) 61:2790. 10.4081/ejh.2017.2790 28735520 PMC5452634

[53] Darlington LG Mackay GM Forrest CM Stoy N George C Stone TW. Altered kynurenine metabolism correlates with infarct volume in stroke. Eur J Neurosci. (2007) 26:2211–21. 10.1111/j.1460-9568.2007.05838.x 17892481

[54] Brouns R Verkerk R Aerts T De Surgeloose D Wauters A Scharpe S. The role of tryptophan catabolism along the kynurenine pathway in acute ischemic stroke. Neurochem Res. (2010) 35:1315–22. 10.1007/s11064-010-0187-2 20490917

[55] Gold AB Herrmann N Swardfager W Black SE Aviv RI Tennen G. The relationship between indoleamine 2,3-dioxygenase activity and post-stroke cognitive impairment. J Neuroinflamm. (2011) 8:17. 10.1186/1742-2094-8-17 21324164 PMC3055827

[56] Bensimon K Herrmann N Swardfager W Yi H Black SE Gao FQ. Kynurenine and depressive symptoms in a poststroke population. Neuropsychiatr Dis Treat. (2014) 10:1827–35. 10.2147/ndt.s65740 25285006 PMC4181733

[57] Mo X Pi L Yang J Xiang Z Tang A. Serum indoleamine 2,3-dioxygenase and kynurenine aminotransferase enzyme activity in patients with ischemic stroke. J Clin Neurosci. (2014) 21:482–6. 10.1016/j.jocn.2013.08.020 24412293

[58] Ormstad H Verkerk R Amthor KF Sandvik L. Activation of the kynurenine pathway in the acute phase of stroke and its role in fatigue and depression following stroke. J Mol Neurosci. (2014) 54:181–7. 10.1007/s12031-014-0272-0 24664436

[59] Goldstein LE Leopold MC Huang X Atwood CS Saunders AJ Hartshorn M. 3-Hydroxykynurenine and 3-hydroxyanthranilic acid generate hydrogen peroxide and promote alpha-crystallin cross-linking by metal ion reduction. Biochemistry. (2000) 39:7266–75. 10.1021/bi992997s 10852726

[60] Leipnitz G Schumacher C Dalcin KB Scussiato K Solano A Funchal C. *In vitro* evidence for an antioxidant role of 3-hydroxykynurenine and 3-hydroxyanthranilic acid in the brain. Neurochem Int. (2007) 50:83–94. 10.1016/j.neuint.2006.04.017 16959377

[61] Albuquerque EX Schwarcz R. Kynurenic acid as an antagonist of alpha7 nicotinic acetylcholine receptors in the brain: facts and challenges. Biochem Pharmacol. (2013) 85:1027–32. 10.1016/j.bcp.2012.12.014 23270993 PMC3721521

[62] Varga N Csapo E Majlath Z Ilisz I Krizbai IA Wilhelm I. Targeting of the kynurenic acid across the blood-brain barrier by core-shell nanoparticles. Eur J Pharm Sci. (2016) 86:67–74. 10.1016/j.ejps.2016.02.012 26924227

[63] Fukui S Schwarcz R Rapoport SI Takada Y Smith QR. Blood-brain barrier transport of kynurenines: implications for brain synthesis and metabolism. J Neurochem. (1991) 56:2007–17. 1827495 10.1111/j.1471-4159.1991.tb03460.x

[64] Robotka H Sas K Agoston M Rozsa E Szenasi G Gigler G. Neuroprotection achieved in the ischaemic rat cortex with L-kynurenine sulphate. Life Sci. (2008) 82:915–9. 10.1016/j.lfs.2008.02.014 18387638

[65] Schwarcz R Whetsell WO Jr Mangano RM. Quinolinic acid: an endogenous metabolite that produces axon-sparing lesions in rat brain. Science. (1983) 219:316–8. 6849138 10.1126/science.6849138

[66] Steiner J Walter M Gos T Guillemin GJ Bernstein HG Sarnyai Z. Severe depression is associated with increased microglial quinolinic acid in subregions of the anterior cingulate gyrus: evidence for an immune-modulated glutamatergic neurotransmission? J Neuroinflammation. (2011) 8:94. 10.1186/1742-2094-8-94 21831269 PMC3177898

[67] Robinson RG Jorge RE Post-stroke depression: a review. Am J Psychiatry. (2016) 1:221–31. 10.1176/appi.ajp 26684921

[68] Phillips RS Iradukunda EC Hughes T Bowen JP. Modulation of enzyme activity in the kynurenine pathway by kynurenine monooxygenase inhibition. Front Mol Biosci. (2019) 6:3. 10.3389/fmolb.2019.00003 30800661 PMC6376250

[69] Kindler J Lim CK Weickert CS Boerrigter D Galletly C Liu D. Dysregulation of kynurenine metabolism is related to proinflammatory cytokines, attention, and prefrontal cortex volume in schizophrenia. Mol Psychiatry. (2019). 10.1038/s41380-019-0401-9. [Epub ahead of print]. 30940904 PMC7577855

[70] Mandi Y Vecsei L. The kynurenine system and immunoregulation. J Neural Transm. (2012) 119:197–209. 10.1007/s00702-011-0681-y 21744051

[71] Lestage J Verrier D Palin K Dantzer R. The enzyme indoleamine 2,3-dioxygenase is induced in the mouse brain in response to peripheral administration of lipopolysaccharide and superantigen. Brain Behav Immun. (2002) 16:596–601. 10.1016/S0889-1591(02)00014-4 12401474

[72] Prendergast GC Malachowski WP DuHadaway JB Muller AJ. Discovery of IDO1 inhibitors: from bench to bedside. Cancer Res. (2017) 77:6795–811. 10.1158/0008-5472.CAN-17-2285 29247038 PMC6021761

[73] Kim T Chelluboina B Chokkalla AK Vemuganti R. Age and sex differences in the pathophysiology of acute CNS injury. Neurochem Int. (2019) 127:22–8. 10.1016/j.neuint.2019.01.012 30654116 PMC6579702

[74] Yu QJ Tao H Wang X Li MC. Targeting brain microvascular endothelial cells: a therapeutic approach to neuroprotection against stroke. Neural Regen Res. (2015) 10:1882–91. 10.4103/1673-5374.170324 26807131 PMC4705808

